# The Involvement of microRNAs in Bone Remodeling Signaling Pathways and Their Role in the Development of Osteoporosis

**DOI:** 10.3390/biology13070505

**Published:** 2024-07-07

**Authors:** Rogelio F. Jiménez-Ortega, Alejandra I. Ortega-Meléndez, Nelly Patiño, Berenice Rivera-Paredez, Alberto Hidalgo-Bravo, Rafael Velázquez-Cruz

**Affiliations:** 1 Laboratorio de Genómica del Metabolismo Óseo, Instituto Nacional de Medicina Genómica (INMEGEN), Mexico City 14610, Mexico; rogeliofrank.jimenez@uneve.edu.mx; 2 Unidad de Acupuntura Humana Rehabilitatoria, Universidad Estatal del Valle de Ecatepec (UNEVE), Ecatepec de Morelos 55210, Mexico; 3 Unidad Académica de Ciencias de la Salud, Universidad ETAC Campus Coacalco, Coacalco de Berriozábal 55700, Mexico; alejandra.ortega@universidadetac.edu.mx; 4 Unidad de Citometría de Flujo (UCiF), Instituto Nacional de Medicina Genómica (INMEGEN), Mexico City 14610, Mexico; lnpatino@inmegen.gob.mx; 5 Centro de Investigación en Políticas, Población y Salud, Facultad de Medicina, Universidad Nacional Autónoma de México, Mexico City 04510, Mexico; bereriverap@comunidad.unam.mx; 6 Departamento de Medicina Genómica, Instituto Nacional de Rehabilitación, Mexico City 14389, Mexico; ahidalgo@inr.gob.mx

**Keywords:** miRNAs, bone metabolism, osteoporosis, bone remodeling, osteoclasts, osteoblasts

## Abstract

**Simple Summary:**

Osteoporosis is a disease that is characterized by bone mass loss and microarchitecture deterioration, due to alterations in the bone remodeling mechanism, which must maintain a balance between bone resorption and formation. In recent years, it has been described that microRNAs (miRNAs) play an essential role in bone activation, differentiation, and homeostasis, since they can act as regulators of genes that participate in different signaling pathways. Therefore, altered expression of distinct miRNAs can affect the pathology of bone diseases such as osteoporosis. This review analyzes the current knowledge on the role of miRNAs in the various signaling pathways that maintain bone homeostasis in humans.

**Abstract:**

Bone remodeling, crucial for maintaining the balance between bone resorption and formation, relies on the coordinated activity of osteoclasts and osteoblasts. During osteoclastogenesis, hematopoietic stem cells (HSCs) differentiate into the osteoclast lineage through the signaling pathways OPG/RANK/RANKL. On the other hand, during osteoblastogenesis, mesenchymal stem cells (MSCs) differentiate into the osteoblast lineage through activation of the signaling pathways TGF-β/BMP/Wnt. Recent studies have shown that bone remodeling is regulated by post-transcriptional mechanisms including microRNAs (miRNAs). miRNAs are small, single-stranded, noncoding RNAs approximately 22 nucleotides in length. miRNAs can regulate virtually all cellular processes through binding to miRNA-response elements (MRE) at the 3’ untranslated region (3′UTR) of the target mRNA. miRNAs are involved in controlling gene expression during osteogenic differentiation through the regulation of key signaling cascades during bone formation and resorption. Alterations of miRNA expression could favor the development of bone disorders, including osteoporosis. This review provides a general description of the miRNAs involved in bone remodeling and their significance in osteoporosis development.

## 1. Introduction

Osteoporosis (OP) is a metabolic disease characterized by low bone mineral density (BMD), leading to increased susceptibility to bone fragility fractures [1]. It is prevalent among adults over 50, and postmenopausal women are particularly affected [2]. OP stems from dysregulated bone remodeling, where osteoclasts remove old or damaged bone, replaced by osteoblasts with new bone [3]. In recent years, OP management strategies have improved considerably. However, the complexity of the multiple molecular mechanisms responsible for the development of OP, and the lack of specific therapeutic targets, have hindered advances in preventing OP [4]. MicroRNAs (miRNAs) have garnered attention for their involvement in various biological processes, including cell differentiation, migration, invasion, and apoptosis, with their dysregulation linked to metabolic diseases, including osteoporosis [5]. miRNAs are a class of small, noncoding RNAs that regulate gene expression post-transcriptionally through mRNA degradation or translation inhibition [6]. Recent studies have shown that miRNAs play an essential role in bone remodeling since they are involved in osteoclast and osteoblast differentiation. Therefore, they have been proposed as potential biomarkers and possible therapeutic targets for treating [7]. This review aims to synthesize the current evidence on miRNAs participating in bone remodeling and their potential as biomarkers for early OP detection.

## 2. Bone Remodeling

**Figure 1:**
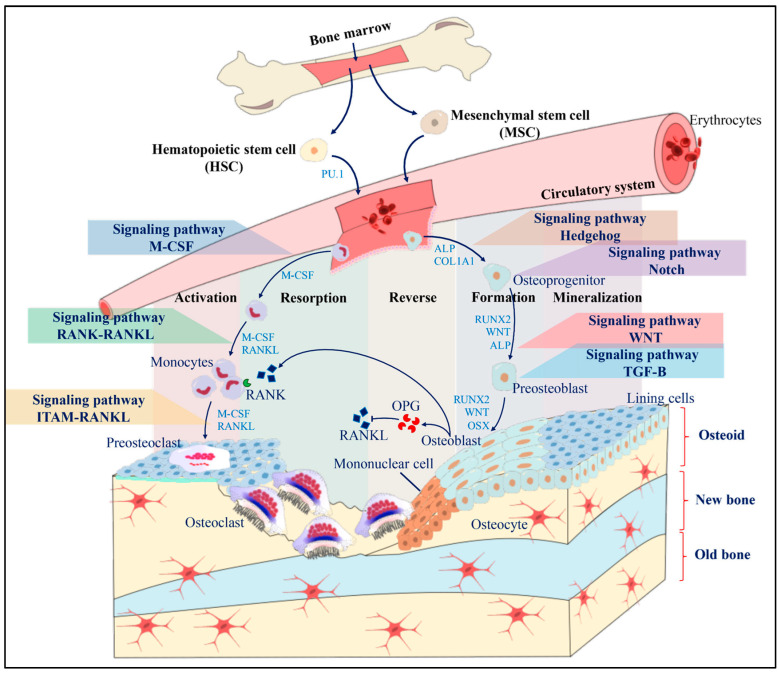
Scheme of a BMU and the mechanism of bone remodeling. Initially, the resting bone surface is covered by lining cells and pre-osteoblasts. Mononuclear cells secrete OPG, which suppresses osteoclastogenesis until the activation, resorption, reverse, formation and mineralization stages are activated. During the differentiation of osteoclasts and osteoblasts, different signaling pathways are activated that allow obtaining mature bone cells capable of performing specific functions. Osteoclastogenesis can be activated through the M-CSF signaling pathways, the RANK-RANKL pathway, and the ITAM RANKL pathway, while osteoclastogenesis is activated through the Hedgehog signaling pathway, the Notch pathway, the Wnt pathway, and the TGF-B pathway.

Bone is a dynamic tissue that is continuously turned over throughout life. Normal bone homeostasis depends on a self-renewal mode named bone remodeling, which is mainly maintained by the balance between osteoclastic bone resorption and osteoblastic bone formation [8]. This process comprises five main stages: activation, resorption, reversion, formation, and termination (Figure 1) [9]. It occurs within the basic multicellular unit (BMU), where osteoblasts (bone tissue-forming cells), osteoclasts (bone tissue resorption cells), osteocytes (mechanosensory and chemotaxis cells), and bone lining cells collaborate harmoniously [10]. The functions of these cell types are precisely controlled through their different intracellular events. The coupling systems regulate them during their interaction, so the regulation of any intracellular event or deterioration in their coupling factors can affect development and bone remodeling. The BMU, covered by bone lining cells, orchestrates resorption and formation processes, maintaining bone volume and responding to mechanical damage. The disruption of this balance leads to mineral diseases like osteopetrosis or osteoporosis [11].

The current data demonstrate the genetic and epigenetic factors that can affect bone remodeling [12]. Epigenetics is defined as the mechanisms regulating gene expression resulting in a determined phenotype, without altering the DNA sequence; furthermore, epigenetic mechanisms can be inherited [13,14]. There are currently three recognized epigenetic mechanisms: histone modification, DNA methylation, and noncoding RNAs (ncRNAs). Among (ncRNAs), microRNAs (miRNAs) have attracted special interest for their role in bone development and function [12]. The miRNAs have been found to be involved in multiple biological processes, including cell differentiation and proliferation [15]. Therefore, numerous miRNAs have been found to be involved in the regulation of bone homeostasis, and they play critical roles in bone remodeling.

## 3. Biogenesis of miRNAs

miRNAs are the most studied noncoding RNAs related to bone metabolism and bone diseases. miRNAs are small ncRNAs ranging from 19 to 25 nucleotides in length that can regulate gene expression post-transcriptionally in eukaryotic organisms. miRNAs genes are transcribed by the RNA pol II/III. The primary transcript is called pri-miRNA. In some cases, several miRNAs from a single locus can be transcribed simultaneously [16,17]; alternatively, miRNAs can be located within an intron or an untranslated region (UTR). There are two main miRNA biogenesis pathways: the canonical and non-canonical [18]. In the canonical pathway, the Drosha/DGCR8 complex and other associated proteins process the pri-miRNA into a miRNA precursor (pre-miRNA), which acquires a hairpin structure of approximately 60–100 nucleotides in length. Pre-miRNA is transported from the nucleus to the cytoplasm through the protein Exportin 5 (XPO5) and Ran-GTP [17].

**Figure 2:**
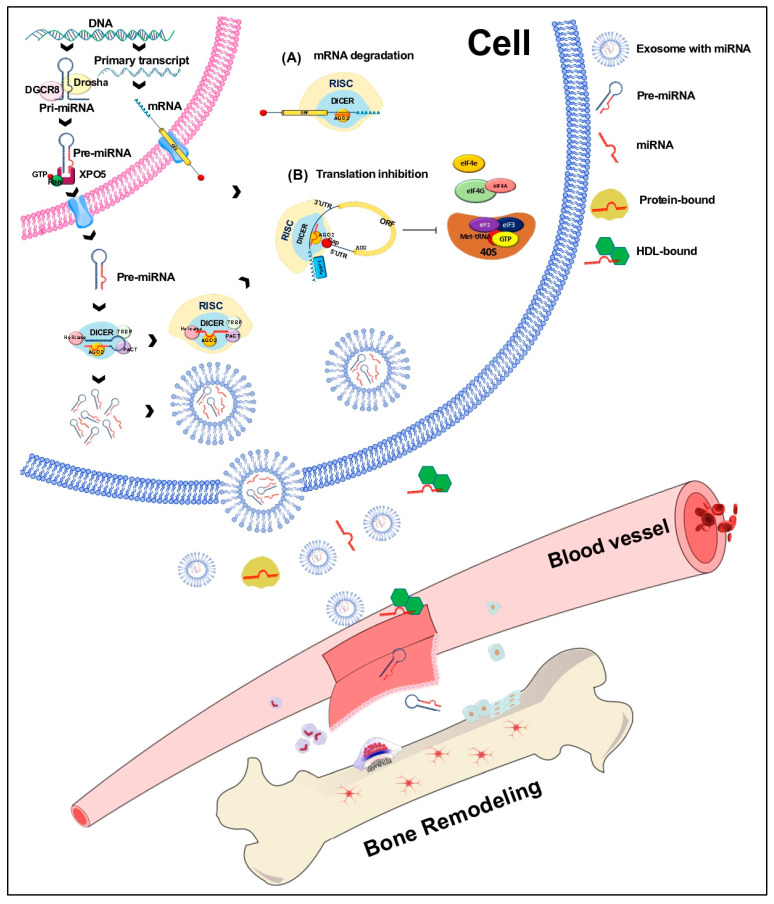
Biogenesis of miRNAs. miRNAs are transcribed by RNA polymerase II or III into primary miRNAs (pri-miRNAs) that are processed by Drosha/DGCR8 into miRNA precursors (pre-miRNAs). The strand of a mature miRNA is shown in red; the pre-miRNA is transported from the nucleus to the cytoplasm by exportin 5 (XPO5), where it is processed by Dicer/TRBP into a duplex miRNA. A helicase unwinds this miRNA, and the mature strand (red) is incorporated into the RNA-induced silencing complex (RISC). Depending on the complementarity of the miRNA with the seed region of a target mRNA, the RISC complex mediates the downregulation of gene expression either by mRNA degradation (A) or by translational repression (B). Circulating miRNAs are produced within the donor cell and are bound to proteins. They are exported directly or packaged into exosomes and released into circulation. miRNAs are also bound to HDL (high-density lipoproteins); however, the binding mechanisms are still under investigation.

Conversely, in the non-canonical pathway, the miRNAs lying on introns, called miRtrons, are processed by the spliceosome and are transported directly to the cytoplasm [19]. Once in the cytoplasm, pre-miRNAs are processed by the Dicer complex to generate a mature miRNA duplex. Subsequently, the RNA-induced silencing complex (RISC) is formed by the Argonaute protein (AGO), the PAK activator protein (PACT), the RNA-binding protein (TRBP), and one of the strands from the mature miRNA duplex. The RISC complex targets mRNA through base pairing between the seed region of the miRNA and the miRNA Response Elements (MREs), located at the 3′UTR of the target mRNA. Perfect base complementarity promotes the AGO2 protein to cleave the mRNA, leading to degradation by endonucleases. When base complementarity is imperfect, a hairpin is formed, promoting the translational suppression of the target mRNA. Furthermore, circulating miRNAs are found in various forms in the bloodstream (Figure 2): 90% of extracellular miRNAs are bound to AGO proteins, while the remaining 10% are packaged into exosomes in apoptotic bodies or bound to high-density lipoproteins (HDL) [20].

## 4. Osteoclastogenesis

Bone remodeling is intricately regulated by a delicate balance between bone resorption and formation [21]. The first phase, termed “activation”, commences in the BMU, where mechanical damage stimulates bone resorption through the release of cytokines responsible for recruiting osteoclast precursors to the bone surface to induce bone decalcification. The macrophage colony-stimulating factor (M-CSF) and the receptor activator nuclear kappa B ligand (RANKL) induce the differentiation of hematopoietic progenitor cells into osteoclast. Under physiological conditions, these cytokines are released by osteoblasts and stromal cells. They are also required for the survival, proliferation, and expression of receptor activator nuclear kappa B (RANK) in osteoclast precursors [22]. RANKL can initiate signaling cascades for osteoclast differentiation, such as those dependent on NF-KB, c-Fos, cell nuclear factor transcription factor t (NFATc1), and microphthalmia-induced transcription factor (MITF) [23]. The differentiated osteoclasts will start with the “resorption” phase, forming a wavy edge that allows them to adhere to the bone surface. Between the osteoclasts and the bone surface, an isolated microenvironment is formed where proton pumps release ions for acidifying the medium, dissolving the mineralized component of the bone matrix and forming a cavity known as Howship’s lagoon. Afterwards, the exposed organic matrix is degraded by cathepsin k, initiating the “reverse” phase [24]. In the reverse phase, mononuclear cells prepare the newly resorbed bone surface and recruit osteoblast precursors, which mature into osteoblasts and constitutively express type I collagen. Osteoblasts trapped within the mineralized matrix continue their differentiation to become osteocytes, while osteoblasts on the surface become lining cells [3].

### 4.1. Signaling Pathways Involved in Osteoclast Differentiation

**Figure 3:**
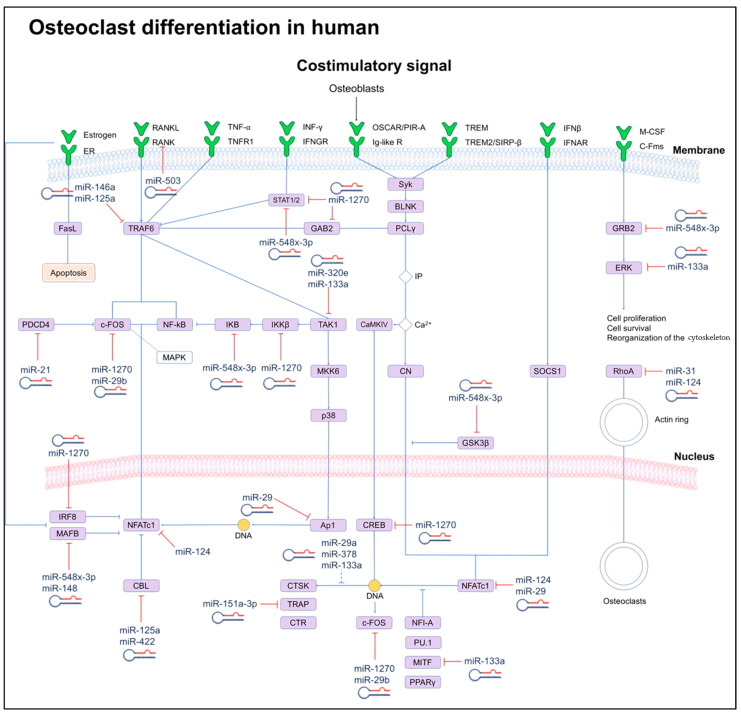
Scheme of signaling networks (OPG/RANK/RANKL) involved in osteoclast differentiation and their regulation induced by miRNAs. miRNAs can directly (solid lines) or indirectly (dotted lines) inhibit key genes for osteoclast differentiation. Dashed lines indicate cellular processes.

The connection between the miRNAs and the involved pathways in osteoclastogenesis is shown in Figure 3 and Table 1. In recent years, several studies have unraveled key signaling mechanisms involved in osteoclast differentiation, including the signaling pathways, such as the M-CSF and RANK ligand (RANKL) pathways, which are critical for bone resorption.

#### 4.1.1. M-CSF Signaling Pathway

Differentiation into osteoclasts begins when M-CSF binds to the c-Fms receptor of a hematopoietic precursor. Signal transduction leads to the activation of the extracellular signal-regulated kinase (ERK) through Grb2 and the phosphoinositide 3-kinase (PI-3K/Akt) [40]. Hematopoietic stem cell differentiation into osteoclasts is induced by the activation of transcription factors such as binding protein 1 (PU.1) and MITF. In hematopoietic stem cells, PU.1 stimulates the expression of CSF1R, the M-CSF receptor.

Another transcription factor that plays an essential role in osteoclastogenesis is the activating protein 1 (AP-1), a member of the Fra, Fos, Jun, and activating transcription factor (ATF) family. On the other hand, the transcription factor MITF is involved in the late stage of osteoclastogenesis through interaction at a mitogen-activated protein kinase (MAPK) consensus site, where M-CSF induces MITF phosphorylation [41]. MITF phosphorylation induces expression of the anti-apoptotic gene of B cell lymphoma 2 (BCL2) and promotes macrophage survival. In addition, MITF and PU.1 can significantly increase RANK promoter activity. On the contrary, MITF levels are regulated by RANKL [42]. In this sense, both PU.1 and MITF have an essential role in the differentiation and survival of osteoclasts by the induction of specific genes [43].

#### 4.1.2. RANKL–RANK Signaling Pathway

Activation of RANKL–RANK pathway induces the expression of genes driving the fusion of cells derived from the monocyte/macrophage lineage, for example, dendritic cell-specific transmembrane protein (CD-STAMP), as well as genes involved in the resorption process of mineral tissue [44], including cathepsin K (CTSK), chloride channel 7 (CIC-7), matrix metalloproteinase 9 (MMP9), and calcitonin receptor (CTR) [45]. The binding of RANKL to RANK induces the recruitment of TRAF-6, which activates the PI3K family of transcription factors, NF-kB, and the MAPK pathways, including ERK, c-Jun N-terminal kinase (JNK), and p38. NF-kB induces the expression of cytokines such as interleukin 6 (IL6), interleukin 1 (IL-1), TNF, and GM-CSF; the p38 protein kinase is activated through phosphorylation of the MAPK kinase (MKK), allowing the downstream activation of MITF. The inhibition of p38 increases ERK phosphorylation while maintaining a balance between ERK and p38 phosphorylation. RANKL induces the expression of the AP-1/c-Fos complex [46]. On the other hand, the expression of NFATc1 depends on the TRAF-6/NF-kB/c-Fos pathways, activated by RANKL and Ca^+2^ signaling. NFATc1 is a transcription factor that regulates osteoclast-specific gene expression such as tartrate-resistant acid phosphatase (TRAP), CTSK, calcitonin receptor (CGRP), osteoclast-associated receptor (OSCAR), and integrin 3 (ITGB3) [47].

#### 4.1.3. Tyrosine-Based Immunoreceptor (ITAM) Signaling Pathway

Recent studies have demonstrated that ITAM adapter proteins are involved in the formation and function of osteoclasts. They signal to activate Syk kinase and PLCγ2 which initiates Ca^2+^ oscillations that can result in activation of the key transcription factor, NFATc1, controlling the differentiation of pre-osteoclasts and multinucleation. The activation of M-CSF/RANKL-induced signaling is not sufficient to complete the osteoclast differentiation process, so it is necessary to activate costimulatory signals dependent on the immunoreceptor tyrosine-based activation motif (ITAM); these signals are activated by multiple immunoreceptors involved in osteoclastogenesis, among them are the Fc receptor subunit (FcR) and DNAX activating protein 12 (DAP12), which are part of ITAM. In osteoclast precursor cells, FcR and DAP12 are associated with multiple immunoreceptors that activate the calcium signaling pathway through phospholipase C (PLC) [48]. These receptors include OSCAR, myeloid cell-activating receptor 2 (TREM-2), signal regulatory protein (SIRP1), and paired Ig-like receptor A (PIR-A), which work together with RANKL and ITAM to costimulate RANK [49]. However, although ITAM adapter signaling is critical for normal bone remodeling, estrogen deficiency induces an ITAM adapter-independent bypass mechanism enhancing osteoclastogenesis and activation in specific bony microenvironments [50].

## 5. The Role of miRNAs in Osteoclastogenesis

In the following sections, we highlight several critical miRNAs playing an essential role in osteoclastogenesis. The have been proposed as potential markers for the early detection of bone-related diseases.

### 5.1. miR-30

This miRNA targets genes associated with stimulation of osteoclastogenesis (IL-8, IL-11), the inhibition of osteoblastogenesis (DKK-1), tumor cell osteomimetics (RUNX2, CDH11), and invasiveness (CTGF, ITGA5, ITGB3) [51]. Abnormal levels of secret antagonists of Wnt signaling can displace bone remodeling in both directions. DKK1 is considered an inhibitor of Wnt/β-catenin signaling, which is linked to new bone formation by functioning as a positive regulator of osteoblasts. Meanwhile, RUNX2 and Wnt/β-catenin signaling plays a crucial role in MSCs migration and osteoblast differentiation [52,53]. Therefore, the miR-30-induced downregulation of DKK1 and RUNX2 could affect the Wnt/β-catenin signaling pathway and, consequently, the differentiation mechanisms of both the osteoclastic and osteoblastic pathways. On the other hand, the CTGF, ITGA5, and ITGB3 genes are regulators of the PI3K signaling pathway [54]. It has been reported that this signaling pathway is involved in processes of the formation, differentiation, and function of osteoclasts [55].

### 5.2. miR-320e

This miRNA has been observed in extracellular vesicles (EVs) derived from patients with ossification of the posterior longitudinal ligament (OPLL). miR-320e promotes the osteoblastic differentiation of mesenchymal stem cells (MSC) and inhibits the osteoclastic differentiation of monocytes. miR-30e targets TGF-β-activated kinase 1 (TAK1), which is an essential activator of osteoclastogenesis [56]. TAK1 is a crucial regulator of innate and proliferative immune signaling pathways such as TNF, IL-1R, and TLR. Its activation phosphorylates the IkB kinase (IKK) complex, p38, JNK, and ERK, leading to the activation of nuclear factor (NF)-κB and the MAPK signaling pathways. Therefore, the regulation of TAK1, induced by miR-320e, could be a critical factor in the regulation of osteoclastogenesis via NF-κB [57].

### 5.3. miR-1270

This miRNA was described as a positive regulator of osteoclastogenesis since it inhibits the expression of interferon regulatory factor 8 (IRF8), which has been described as an anti-osteoclastogenic gene. The positive regulation of this miRNA promotes monocyte/osteoclast differentiation in postmenopausal women, favoring the development of osteoporosis [58]; therefore, this miRNA could be a potential biomarker candidate for the early detection of osteoporosis [59]. It has been reported that the RANK–RANKL signaling pathway activates the NF-κB and c-Fos signaling, which in turn activates the master transcription factor NFATc1, which promotes osteoclastogenesis. However, it has been reported that the activation of factors such as IRF8, MAFB, and IDS can suppress osteoclast differentiation [60]. IRF8 is regulated by miR-1270 in circulating monocytes from postmenopausal women, so this miRNA could stimulate osteoclast differentiation through the RANK–RANKL signaling pathway.

During the process of differentiation and maturation of osteoclasts, other changes in the expression of miRNAs have been described in humans. However, these miRNAs’ molecular mechanisms and functional roles are still unclear. Table 1 describes the role of the most relevant miRNAs [25,26,27,28,29,30,31,32,33,34,35,36,37,38,39] in osteoclastogenesis that have been described in recent years.

## 6. Osteoblastogenesis

**Figure 4:**
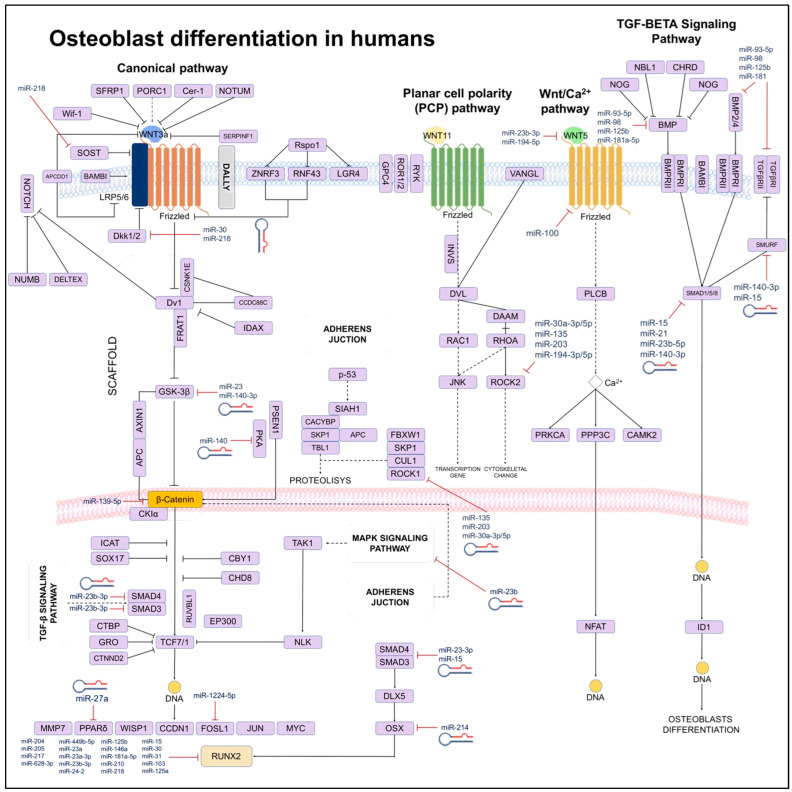
Scheme of signaling networks (TGF-β/BMP/Wnt) involved in osteoblast differentiation. It is show that miRNAs can directly (solid lines) or indirectly (dotted lines) to inhibit genes regulating differentiation processes, apoptosis, or the phenotype of osteoblasts.

Bone formation begins with MSCs, derived from the mesoderm in the early stages of embryonic development. The differentiation of MSC to osteoblasts is crucial for maintaining bone remodeling. Osteoblasts secrete organic bone matrix, promote bone matrix mineralization, and maintain bone homeostasis. Osteoblast dysfunction can lead to the destruction of bone microarchitecture and defects in bone formation, leading to the development of metabolic diseases such as osteoarthritis and osteoporosis (Figure 4).

### 6.1. Signaling Pathways Involved in Osteoblast Differentiation

Osteogenesis is regulated by different signaling pathways, such as, M-CSF, and RANKL, involving several transcription factors, like *RUNX2*, the nuclear factor of acti-vated T cells (NFAT), and Osterix (Osx). *RUNX2* is the determinant transcription; its inactivation can inhibit or delay osteoblast formation. *RUNX2* is a critical factor in osteoblast-mediated bone formation and requires precise regulation by mechanisms such as Osx [61]. Additional pathways are also involved in the osteoblast differentiation process and the regulation of the ossification process, such as the Hedgehog pathway (Hh), the Wnt pathway, the BMP pathway, and the Notch pathway [62].

#### 6.1.1. Wnt Signaling Pathway

The Wnt family comprises a set of highly conserved genes that regulate cell behavior, expression, adhesion, and polarity. The Wnt-induced signaling was thought to be mediated by β-catenin. However, current evidence supports that the Wnt pathway activates β-catenin-independent mechanisms by what is known as the “non-canonical pathway” to regulate vertebrate development [63]. Just as the canonical pathway is essential in vertebrate development and bone diseases, the non-canonical Wnt pathway is involved in bone formation. The non-canonical pathway is divided into two main sub-pathways: the Wnt–planar-cell-polarity pathway (Wnt–PCP pathway) and the Wnt–calcium pathway (Wnt–Ca^2+^ pathway). The Wnt5a protein regulates limb morphogenesis, chondrogenesis, and osteoblastogenesis through the receptor tyrosine kinase (Ror) proteins in the Wnt–PCP pathway. Furthermore, this pathway also regulates osteoclastogenesis. The Wnt5a-Ror2 signaling activates the expression of JNK, which is responsible for recruiting c-Jun from promoting the expression of RANK on the surface of osteoclast precursor cells. On the other hand, in the Wnt–Ca^2+^ pathway, the Wnt5a ligand binds to the Frizzled receptor, triggering an increase of inositol 1,4,5-trisphosphate (IP3), 1,2-diacylglycerol (DAC), and Ca^2+^ with PLC. This interaction triggers the activation of the NF-kB signaling pathway and the expression of transcription factors such as NFAT, which regulates osteoclastogenesis [64]. Different studies have reported that the Wnt–Lrp5 interaction can induce mTORC2-AKT signaling activity and triggers glycolytic enzymes in bone cells to promote bone formation. These findings indicate that Wnt signaling can regulate bone homeostasis [65].

#### 6.1.2. Ligands and Agonists of the Wnt Pathway in Bone

Wnt ligands are cysteine-rich proteins that contain an N-terminal signal peptide for secretion and have effects at various stages of bone development, including chondro-genesis, osteoclastogenesis and osteoblastogenesis. Mutations of the Wnt1 gene have been found in children with osteogenesis imperfecta, which is a disorder characterized by increased bone fragility and other connective tissue manifestations [66]. Wnt3a regulates the fate of the dorsal mesoderm and is required during the early stages of limb formation and craniofacial development [67]. In a mouse model of R26floxneo Wnt4 with a Col2a1-Cre mutation, the conditional expression of Wnt4 results in dwarfism and an increased number of hypertrophic chondrocytes. In addition, it was also observed that Wnt5a and Wnt5b activate the proliferation and differentiation of chondrocytes [68]. In different studies with mouse models, it was observed that Wnt3a +/− and Wnt5a +/− presented a low bone mass phenotype [69]. Wnt6, Wnt10a, and Wnt10b have also been reported to stimulate osteo-blastogenesis and inhibit adipogenesis [70].

Mutations in the ligands Wnt7 and Wnt11 activate the differentiation of chondrocytes and osteoblasts, while deficiency of Wnt16 decreases BMD and increases fracture risk [71]. The current data report that parathyroid hormone (PTH) influences Wnt signaling at different stages of bone development. PTH decreases the expression of Wnt inhibitors such as Sost, leading to an increase of Wnt signaling. In a study by Tian Y. et al. 2011, in a model of MC3T. E1 osteoblasts treated with PTH, the characteristic markers of osteoblast differentiation were blocked by decreasing B-catenin expression [72]. On the other hand, transgenic mice expressing a constitutively active PTH receptor in osteocytes showed an increase in the number of osteoblasts and bone mass and the downregulation of Sost [73]. In postmenopausal women, PTH can reduce circulating sclerostin levels. The effects of PTH on the canonical Wnt signaling pathway can upregulate FZD-1 or LRP6 receptor complex proteins and decrease the Dkk-1 antagonist [74]. Although PTH treatment reduces Dkk-1 expression, Dkk-1 upregulation does not inhibit the anabolic response to PTH in vivo [75]. Evidence suggests that in both in vivo and in vitro models, there is a direct crosstalk between PTH1R and the Wnt signaling pathway. PTH binding to the PTHR receptor induces the association of the PTH-PTH1R complex with the extracellular domain of the Lrp6/Wnt receptor in the absence of the Wnt ligand, resulting in the phosphorylation of Rlp6, [76]. These findings suggest that PTH induces osteoblast differentiation by activating the canonical pathway of Wnt.

#### 6.1.3. Notch Signaling Pathway

The Notch signaling pathway requires cell-to-cell interaction. It is initiated when Jagged ligands 1/2 (JAG) and delta-like ligands 1/3/4 (DLL) interact with Notch receptors (1-4 in mammals). Ligand binding leads to the proteolytic cleavage of the Notch receptor by the action of the γ-secretase complex, releasing the Notch intracellular domain (NICD). The NICD translocates to the nucleus and interacts with the DNA-binding protein RBPjk/CBF1, displacing corepressors and allowing the assembly of an activator complex that includes transcriptional coactivators similar to the Mastermind protein type 1 (MAML1), which is involved in cell differentiation. Notch target genes include the Hairy transcription receptor (HES-1) and HES associated with the YRPW-like motif (HEY) [77]. The Notch signaling pathway interacts with the Wnt signaling pathway and bone morphogenic protein (BMP) to regulate skeletal development and homeostasis. In addition, in vitro experiments have showed that NICD overexpression opposes the differentiation of osteoblasts induced by exogenous Wnt. This effect is regulated by HES-1 [78]. Other in vitro studies investigating the relationship between Notch signaling and BMP report opposite results, showing that NICD overexpression blocks the differentiation of osteoblast precursors in the presence of BMP2 [79]. In summary, the signaling induced by Notch enhances osteoblast precursor differentiation [80], whereas Notch inhibition impairs BMP2-induced osteoblast differentiation [81].

#### 6.1.4. Notch Signaling Pathway in Osteoblastogenesis

Studies in mouse models suggest an essential role of the Notch signaling pathway in skeletal development. Knockdown of Notch-1 and Notch-2 in skeletal mesenchymal tissue led to the accumulation of bone tissue within the medullary canal and a severe reduction in trabecular bone mass [82]. These observations suggest that Notch signaling is responsible for suppressing the differentiation of mesenchymal progenitor cells into osteoblasts. Later, it was reported that Notch-2 has a predominant role in suppressing osteoblastogenesis, mainly mediated by Notch-1 and RBPjk13. It has also been observed that downstream of RBPjk, the HEY family of transcriptional suppressors is responsible for the inhibition of RUNX2 activity and the suppression of NFATc1 [83]. In murine models, it has been observed that the Hey transcription factor plays an important role in osteoblastogenesis. Mice expressing Hey present an osteopenic phenotype, while those expressing RBPjk do not show this phenotype. It seems that the suppressive role of Notch-RBPjk signaling in osteoblastogenesis is limited to the early stages of osteoblast differentiation [84]. Studies in transgenic mice overexpressing NICD have revealed different effects depending on the differentiation stage of the osteoblasts. The overexpression of NICD in osteoblastic cells, under the control of the constitutive COL1A1 promoter, generates a phenotype with a high bone mass, but with an excess of immature bone tissue and fibrotic cells in the bone marrow [85]. The expression of NICD stimulates cell proliferation of the osteoblastic lineage in the early stages but prevents the differentiation of mature osteoblasts [86].

#### 6.1.5. TGF-β Signaling Pathway in Osteoblastogenesis

Signaling induced by transforming growth factor-beta (TGF-β) and BMP plays a fundamental role in embryonic skeletal development and bone homeostasis. TGF-β and BMPs act as a tetrameric receptor complex. They are responsible for transducing signals in both the Smad-dependent canonical signaling pathway and the Smad-independent non-canonical signaling pathway. Activated mitogen p38/p38 MAPK is activated to regulate the differentiation of mesenchymal stem cells during skeletal development, bone formation, and bone homeostasis [87]. The transforming growth factors bind to a tetrameric receptor complex comprising two TGF-β receptors of the type I (TβRI/ALK5) and two type II receptor kinases (TβRII) [88]. Transphosphorylases TβRII are responsible for phosphorylating the Smad proteins, activated by Smad 2/3 receptors that subsequently interact with Smad and Smad 4, that are translocated to the nucleus, where they recruit cofactors for gene regulation, such as Creb-binding protein (CBP) and p300 [89].

On the other hand, it has been reported that TGF-β activates a group of R-Smad receptors (Smad1/5/8) through binding to ALK. In addition, it has also been observed that as an alternative pathway, not dependent on Smad, TGF-β activates the protein kinase 1 (TAK1) and the TAK1 binding protein (TAB1), allowing the activation of the p-38/MAPK signaling cascade [89]. TGF-β isoforms are expressed in the perichondrium, periosteum, and epiphyseal growth plate. Mouse models with an attenuated Tgfb2 expression showed severe skeletal abnormalities in both endochondral and intramembranous bone. Meanwhile, Tgfb1- and Tgfb2-deficient mice had no defects. Therefore, it can be assumed that TGF-β1 and TGF-β3 are not essential for the development of the embryonic skeleton. On the contrary, TGF-β2 is necessary for the maintenance of postnatal bone mass by coupling bone resorption and formation. TGF-β proteins are synthesized as precursor molecules containing the latency-associated protein (LAP), which remains non-covalently bound to TGF-β. The cleavage of LAP, induced by bone resorption, allows the release of active TGF-β1, which induces the enrichment of osteoprogenitor cells in the Howship’s cavities [90]. These observations support that the BMP and TGF-β signaling pathways play an essential role in skeletal development and bone homeostasis by interacting with other signaling pathways previously discussed, such as Wnt, Hedgehog, and Notch.

## 7. The Role of miRNAs in Osteoblastogenesis

The osteoblasts cells are involved in bone formation and, thus, maintain homeostasis and metabolism. These cells also play an important role in the development of osteoporosis. Some miRNAs that play an essential role in osteoblastogenesis and bone-related diseases are described below.

### 7.1. miR-23b-3p/miR-885, miR-140-3p and miR-885

In a study carried out by Ramírez et al., the miRNAs miR-23b-3p, miR-140-3p, and miR-885 showed differential expression in the serum of postmenopausal women with low BMD or with osteoporotic fracture, compared to a group with normal BMD. The bioinformatic analysis revealed that these miRNAs regulate genes involved in the Wnt, MAPK, and TGF-β signaling pathways. The authors suggested that these miRNAs could be associated with variation in BMD and highlighted their potential as biomarkers for the early detection of osteoporosis [91].

### 7.2. miR-29-3p, miR-324-3p, and miR-550a-3p

These miRNAs showed a significant correlation with histomorphometric parameters of bone formation and microarchitectural parameters. In addition, these miRNAs were downregulated in patients treated with antiresorptive therapy. The authors proposed these three miRNAs as potential biomarkers for the diagnosis of osteoporosis [92]. Although the potential targets of miR-29b-3p are not mentioned, this miRNA is involved in osteoclast differentiation and bone resorption, mainly at the phase of differentiation from monocyte precursor [93]. miR-324-3p regulates genes such as BSP, RUNX2, OCN, and ALP, which are key in osteoblast differentiation and participate in the Wnt signaling pathway. On the other hand, miR-324-3p can also promote osteoblastogenesis through the regulation of SMAD7 [94]. The miRNA miR-550a-3p was identified in the serum of postmenopausal women and has been suggested as a potential biomarker for the early diagnosis of osteoporosis. This miRNA targets osteocalcin (OCN), which is a crucial participant in the Wnt signaling pathway [95].

### 7.3. miR-30a-3p/5p, miR-194-3p/5p, miR-27b-3p/5p and miR-34a-3p/5p

In a work published by Zhou et al., miRNAs and differentially expressed genes (DEGs) were identified in the serum of a group of postmenopausal women with both high and low BMD. The authors identify 34 miRNAs, of which hsa-miR-30a-3p/5p, hsa-miR-194-3p/5p, hsa-miR-27b-3p/5p, and hsa-miR-34a-3p/5p were downregulated in the samples from women with osteoporosis compared to a control group. The authors propose that the expression of these miRNAs could be related to the suppression of osteoclast survival and the promotion of osteoblast activity. Therefore, the authors suggested that these miRNAs could play a key role in regulating bone formation since the overexpression of miR-30a suppress TGFB1, which in turn regulates RUNX2, a transcription factor specific to osteoblasts and bone formation [96].

### 7.4. miR-194

A previous study analyzing blood samples from postmenopausal women with osteopenia and osteoporosis revealed the overexpression of miR-194-5p, hsa-miR-454-3p, hsa-miR-151a-3p, and hsa-miR-590-5p and downregulation of hsa-miR-574-3p, hsa-miR-3907, hsa-miR-4767, and hsa-miR-1972 [97]. From these miRNAs, hsa-miR-194 and hsa-miR-590-5p have been related to bone metabolism, mainly in osteogenesis. They promote osteoblast differentiation through the regulation of the SMAD family genes and RUNX2, which participate in the STAT1 signaling pathway and the Wnt pathway [98,99].

### 7.5. miR-1224-5p

This miRNA was identified as downregulated in plasma derived from patients with osteoporotic fractures, suggesting that it plays a role in osteoclastogenesis. Experimental data showed that expression of miR-1224-5p was positively correlated with the progression of fracture healing. The authors observed that miR-1224-5p slowed down osteoclast differentiation induced by RANKL. Additionally, the expression of miR-1224-5p promoted osteoblast differentiation through the Rap1 signaling pathway, by regulating the ADCY2 gene. Furthermore, the in vivo overexpression of miR-1224-5p significantly promoted fracture healing and facilitated the progression of osteoporosis caused by estrogen deficiency or aging. These results suggest that miR-1224-5p is a critical regulator of osteogenesis and may be a potential therapeutic target for osteoporosis and fragility fractures [100]. Table 2 [28,91,101,102,103,104,105,106,107,108,109,110,111,112,113,114,115,116,117,118,119,120,121,122,123,124,125,126,127,128,129,130,131,132,133,134,135,136,137] describes the function of the most relevant miRNAs involved in osteoblastogenesis that have been described in recent years. Beyond the osteoclastogenesis and osteoblastogenesis pathways, several pathways interact and participate in the bone remodeling process [138].

## 8. miRNAs Involved in Osteocyte Differentiation

Osteocytes are the last level of differentiation of osteoblasts and are surrounded by the mineralized bone matrix. They act as sensors of chemical signals and mechanical loads that control bone activity through communication with cell effectors that participate in bone remodeling [139]. Osteocytes secrete different molecules that can modulate the function of osteoclasts and osteoblasts on the bone surface, in addition to the mineralized bone matrix and cells of other tissues and organs. Their differentiation involves changes in their morphology that allow them to develop numerous cytoplasmic projections and changes in miRNA expression profiles. Recent studies have reported that osteocytes can release extracellular vesicles containing miRNAs, which can affect the function of skeletal muscle and adipose tissue [140]. It has been shown that miRNAs induce or inhibit the differentiation of osteoblastic cells derived from MSC [141] while, in cell lines, as well as in primary osteocytes, the role of miRNAs in osteocyte differentiation processes has been demonstrated and summarize in Table 3 [142,143,144,145,146,147,148,149,150]. These miRNAs involved in the differentiation of bone cells are known as osteomiR and participate in the differentiation of terminal osteoblasts through the regulation of osteocytic genes [151]. However, as cells progress in their differentiation pathway, they lose the ability to express miRNAs that regulate the activation or inhibition of differentiation, which is why they have been proposed as characteristic markers of osteocytes known as Snord85. In contrast, others are considered negative markers; among them are miR-101a, miR-10a, and the let-7 family, as well as other members of the miR-30 family that increase their expression during late osteocyte differentiation and suppress genes that are important for osteoblast differentiation, such as *Runx2*, *Smad1*/2, and *CCN3* [152]. The role of miRNAs in osteocytes is a field that has been little *explored*, so new research is necessary to determine how miRNAs modulate osteocyte differentiation and participate in bone remodeling.

## 9. The Role of miRNA in Osteoporosis

Bone metabolism is a multifactorial process that involves different types of cells, such as mesenchymal stem cells, hematopoietic stem cells, osteoclasts, osteoblasts, and osteocytes, in which different biological processes, such as proliferation, angiogenesis, differentiation, migration, and apoptosis, are developed, which are regulated by miRNAs [144]. Alterations in the expression profiles of miRNAs can affect the functions of osteoclasts, osteoblasts, and osteocytes, with a consequent imbalance in bone remodeling, leading to the development of OP. However, miRNAs not only regulate bone remodeling but also participate in fracture repair. The fracture repair process consists of several interdependent stages: hematoma formation, inflammation, osteogenesis, chondrogenesis, endochondral ossification, and remodeling, where miRNAs miR-21, miR-140, miR-214 have been proposed as potential biomarkers for monitoring the overall fracture healing process [153]. On the other hand, it has been reported that the blood serum is an ideal sample for the identification of different miRNAs that can be used as biomarkers for the detection of OP, and miR-365a-3p is upregulated in the blood serum. In patients with OP, the levels gradually decrease as osteoinduction in MSC is prolonged, while the downregulation of miR-365a-3p reduces the expression of osteocalcin (OCN), RUNX2, Osteopontin (OPN), and COL1A1, which reduces the bone formation potential [154]. Other studies have reported that miR-579-3p regulates the expression of sirtuin 1 (Sirt1), which is highly expressed in MSCs and is associated with the maintenance of bone homeostasis, so its regulation, induced by miR-579-3p, inhibits the differentiation of MSC into osteogenic cells [155]. Another miRNA identified as upregulated in the blood serum and MSCs of OP patients is miR-96, which was associated with the decreased osteogenic differentiation of MSCs. At the same time, the inhibition of miR-96 mitigated bone loss caused by aging [156]. These studies have demonstrated the presence of miRNAs, in the serum of patients with OP, which show variable expression and can act as potential biomarkers since they act on MSCs, osteoblasts, osteoclasts, and other bone cells. However, using miRNAs as biomarkers may have some disadvantages, such as the lack of standardization during the selection of circulating miRNAs, their identification in a large population, the lack of exhaustive studies on specific diseases, and the effect of confounding variables such as age, sex, and the lack of standards on accurately identifying the origin of miRNAs [157]. Table 4 [106,158,159,160,161,162,163,164,165,166,167,168] shows the miRNAs identified in humans that are involved in OP.

## 10. Effect of Drugs or Biomaterials in the Expression of miRNAs

### 10.1. Bisphosphonates in the Treatment of OP and Changes in the Expression of miRNAs

Current drug treatments for postmenopausal OP (PMO) aim to prevent fractures by inhibiting bone resorption and stimulating bone formation. Oral bisphosphonates (BPs) are a first-line treatment for postmenopausal OP and are well-effective in the prevention of fragility fractures; these drugs decrease bone turnover by inhibiting osteoclast function [169].

Still, few studies have examined the effect of antiosteoporotic treatment on miRNA expression.

Previous studies [170] have investigated the differential expression of miRNAs in previously treatment-naïve women with PMO who were treated with either the potent antiresorptive agent denosumab (Dmab) or the osteoanabolic agent teriparatide (TPTD). The authors reported that while Dmab did not change the relative serum expression of the analyzed miRNAs, TPTD affected the expression of miRNAs that are related to the critical genes regulating osteoblastogenesis, that is, RUNX2 and DKK-1 [170], during the first year of treatment. In a subsequent study, the circulating miRNA expression profile of women with PMO who had received sequential antiosteoporotic treatments was analyzed. The authors observed that circulating miRNAs are differentially affected by treatment with TPTD and Dmab. The TPTD treatment potentially affects the expression of the pro-osteoclastogenic miR-21a-5p and the miRNAs (miR23a-3p, miR-29a-3p, and miR-2861), related to the key osteoblastic genes RUNX2, COL1, and HDAC5, while progressive treatment with Dmab acts in the opposite direction [171]. Recent research by Lia et al. has shed light on the practical implications of miRNA expression in postmenopausal osteoporosis treatment. They found that miR-30a-5p was significantly increased in patients undergoing long-term BP treatment for post-menopausal OP. This miRNA was found to be negatively correlated with bone formation, suggesting that it could serve as a novel mediator of long-term BP treatment that regulates bone formation. The mechanism of action was found to be a direct targeting of RUNX1 to inhibit osteoblastic differentiation in postmenopausal OP patients [172].

Furthermore, the potential of miRNA expression in monitoring treatment response was highlighted in a study on two years of denosumab (DMAB) treatment. The study revealed an upregulation of 7 miRNAs (miR-101-3p, miR-191-5p, miR-26b-5p, miR-32-5p, miR-4508, miR-454-3p, and miR-584-5p) after the treatment period. Four of these miRNAs were primarily expressed in monocytes, indicating a significant impact of DMAB on circulating osteoclast precursor cells. Notably, miR-454-3p, miR-26b-5p, and miR-584-5p emerged as the top biomarker candidates, showing the strongest association with the persistent effect of denosumab on bone in osteoporotic patients. These changes were also associated with BMD gain, further highlighting the potential of miRNA expression in monitoring DMAB treatment response [173]. Additional studies are necessary to confirm circulating miRNAs in serum as reliable indicators of the efficacy of sequential treatment in osteoporosis.

### 10.2. Effect of Biomaterials in the Expression of miRNAs

In recent years, with the increase in studies on miRNAs, the concepts related to biomaterials for repairing bone defects and restoring bone functions have expanded. Previous research focused on different materials and constructs that induce different miRNAs in the bone formation process, such as inorganic silicate-based PerioGlas, which act to alter osteoblast activity due to the exposure of different cell-binding domains and miRNA translations [132,174,175].

Lithium (Li) is a metal with critical therapeutic properties. This metal can be integrated into the structure of various biomaterials. Li-doped bioceramics (capable of immunomodulation), lithium-doped bioactive-glass nanoparticles, and gelatin/PRGF have been used extensively for bone and tooth regeneration. Combining bioactive glass as a bone-inducing mineral phase, gelatin as a biocompatible polymer, PRGF as a source of growth factor, and lithium-ion as a promoting bone repair agent can be considered as an approach to repair bone lesions, and may be helpful as functional bone tissue engineering materials using miRNA as an osteogenic factor. [176,177]. More recently, it has been reported that platelets release growth factors that can modify the expression profiles of miRNAs, and although platelets cannot transcribe a gene, megakaryocytes direct the translational and post-transcriptional regulation of mRNAs and ncRNAs, including miRNAs in bone metabolism cells, so that platelet activation can induce changes in the expression profiles of miRNAs and influence the function of osteoblasts and osteoclasts [178].

## 11. Conclusions

Bone remodeling is a balanced process between bone resorption and formation. However, this process is complicated and involves a large number of signaling pathways and modifications to the expression of regulatory molecules such as miRNAs. miRNAs are associated with numerous biological functions at different stages of bone remodeling due to their capacity to control gene expression, and they can regulate osteoclast and osteoblast differentiation, affecting bone formation and resorption. Therefore, alterations in the expression profiles of miRNAs can affect cell function, leading to bone diseases such as OP. The origin of OP is centered on the excessive activity of osteoclasts; therefore, understanding osteoclast differentiation and maturation has allowed to learn the molecular mechanisms leading to the development of OP and to propose new treatment strategies, based on these molecules. Novel knowledge will support the discovery of molecules with clinical utility as biomarkers for early detection and as therapeutic targets for bone disorders, including OP. The identification of precise and reliable biomarkers for early detection of OP is crucial for intervening in the initial stages of the disease, potentially enabling more effective therapeutic interventions and ultimately improving clinical outcomes and patients’ quality of life. Moreover, understanding how specific molecules, such as miRNAs, regulate the cellular processes involved in OP may lead to the identification of more effective therapeutic targets. miRNAs and other regulatory molecules could offer opportunities to develop highly specific therapeutic approaches that address the underlying causes of the disease rather than merely treating the symptoms. These approaches may include therapies aimed at restoring the balance between bone formation and resorption, which could have a significant impact on the prevention and treatment of OP.

As molecules that can regulate the differentiation of osteoblasts and osteoclasts through multiple pathways, miRNAs can be used as therapeutic agents, because of their ability to regulate the translation of several proteins that are involved in a particular gene’s expression by choosing a variety of methods like degradation or deadenylation of the mRNAs [176]. It may be possible to treat osteoporosis by adjusting their content and expression levels in patients. In this sense, the understanding of miRNAs is still in the initial stages, and studies focused on achieving safety, efficacy, and specific administration systems that allow using a miRNA as a modulator are necessary. Therefore, advancements in the understanding of molecules involved in the pathophysiology of OP will not only provide tools for earlier and more accurate detection of the disease but also open new avenues for the development of more effective and personalized therapies. However, further research is needed to validate these findings and translate them into practical clinical applications that benefit patients with bone disorders. In this context, studying the biology of HSCs and MSCs is necessary to understand the complex interactions between miRNAs and other types of noncoding RNA (ncRNA) as well as their target genes.

## Figures and Tables

**Table 1:** Summary of miRNAs in humans and their targets, expressions, and effects on osteoclast differentiation.

miRNA ID	Study Model	Target Genes	Effects on Bone Remodeling	Reference
miR-31 ↑	BMM	*RhoA* ↓	Promotes osteoclastogenesis	[25]
miR-125 ↑	Osteoblasts	*PRDM1* ↓	Osteoclastogenic inhibition	[26]
miR-125a ↑	PBMCs	*TRAF6* ↓	Suppresses osteoclastogenesis	[27]
miR-21 ↑ miR-125b ↑ miR-122 ↑ miR-124 ↑ miR-148a ↑	Serum samples from patients with osteoporosis	*PDCD4* ↓ *TRAF6* ↓ *RANKL* ↓ *NFATc1* ↓ *MAFB* ↓	Inhibits osteoclastogenesis	[28]
miR-29b ↑	Human	c-*FOS*, *MMP2* ↓	Inhibits osteoclastogenesis	[29]
miR-133a ↑	Human	*CXCL11*, *CXCR3*, *SLC39A1*↓	Not mentioned	[30]
miR-148 ↑	Human	*MAFB* ↓	Enhances osteoclasts differentiation	[31]
miR-151a-3p ↑	Serum samples from patients with osteoporosis	*TRAP*, *cFOS*, *NFATc1* ↓	Regulates osteoclastogenesis	[32]
miR-182 ↑	BMMs	*MX1*, *IFIT2*, *IRF7*, *CXCL10* ↓	Positive regulation of osteoclastogenesis	[33]
miR-214 ↑	BMMs	*ATF4* ↓	Positive regulation of osteoclastogenesis and inhibits bone formation	[34]
miR-320e ↑	Extracellular vesicles from patients with heterotopic ossification of the posterior longitudinal ligament	*TAK1* ↓	Inhibits osteoclastogenesis	[35]
miR-422 ↑	Human	*CBL*, *CD226*, *IGF1*, *PAG1*, *TOB2* ↓	Postmenopausal osteoporosis	[36]
miR-503 ↑	Human	*RANK* ↓	Inhibits RANKL-induced osteoclast differentiation	[37]
miR-548x-3p ↑	Human monocytes	*MAFB*, *STAT1* ↓	Attenuates the proliferative capacity of osteoblastic cells and could promote osteoclastogenesis cell lines	[38]
miR-1270 ↑	Human monocytes Saos-2 and U2-OS osteoblast cell lines	*IRF8* ↓	Attenuates the proliferative capacity of osteoblastic cells and could promote osteoclastogenesis	[39]

↑ upregulated; ↓ downregulated; BMM: bone marrow-derived macrophages. PBMC: peripherical blood mononuclear cells.

**Table 2:** Summary of miRNAs, study models, and miRNAs’ targets, expressions, and effects on osteoblast differentiation.

miRNA ID	Study Model	Target Genes	Effects on Bone Remodeling	Reference
miR-21 ↑ miR-23a-3p ↑ miR-24-3p ↑	Serum samples from patients with osteoporosis	*SMAD7*, *SRPY* ↓ *RUNX2* ↓ *SATB2* ↓	Suppresses osteoblast differentiation	[28]
miR-23b-3p ↑ miR-140-3p ↑	Human blood serum samples	*GSK3B*, *WNT5B*, *RUNX2*, *AKT1*, *AKT2*, *AKT3*, *BMP2*, *SMAD3* ↓ *SMAD2*, *SMAD4*, *MAPK10*, *MAPK14*, *MAPKAPK2*, *SOS1*, *TRAF6*, *TGFB2*, *SMURF1*, *SP1*, *THBS1* ↓	Regulates *Wnt*, *MAPK*, and *TGF*-B signaling pathways	[91]
Let-7 ↑	Mesenchymal stem cells (MSCs)	*HMGA2* ↓	In vivo upregulation induces bone formation by reducing HMGA2 expression	[101]
miR-7 ↑	Primary human osteoblast cells	*EGFR* ↓	Promoting osteoblast apoptosis induced by DEX	[102]
miR-15 ↑	hBMSCs	*RUNX2*, *SMAD7*, *CRIM1* and *SMURF1* ↓	Promotes osteoblast differentiation	[103]
miR-15b ↑	hBMSCs	*USP7* ↓	Suppressing autophagy and differentiation	[104]
miR-30 ↑	Breast cancer cell lines: MDA-B02, MDA-MB-231, T-47, MCF-7, BT-474, ZR-751, SK-BR3, and Hs-578T	*CDH11*, *ITGA5*, *ITGB3*, *RUNX2*, *CTGF*, *DKK1* ↓	Promotes osteoblastogenesis	[105]
miR-31 ↑	hBMSCs	*SATB2*, *RUNX2* ↓	Overexpression of miR-31 significantly reduces the expression of osteogenic transcription factors	[106]
miR-93-5p ↑	Patients with TIONFH	BMP2 ↓	Regulates mechanisms of osteogenic differentiation	[107]
miR-98 ↑	hMSC	BMP2 ↓	Regulates mechanisms of osteogenic differentiation	[108]
miR-100 ↑	hBMSCs	*FZD5*, *FZD8* ↓	Suppresses osteoblast differentiation	[109]
miR-103 ↑	Not mentioned	*RUNX2* ↓	Suppresses osteoblast differentiation	[110]
miR-125a ↑	hADSC cells	*RUNX2*, *ALP*, *OCN*, *VEGF* ↓	Promotes osteogenesis of hADSC mesenchymal stem cells	[111]
miR-125b ↑	Total blood mononuclear cells	*BMP2*, *RUNX2*, *TRAF6*↓	Suppresses osteoblast differentiation	[112]
miR-138 ↑	hMSC	*PTK2*, *FAK* ↓	Suppresses osteoblast differentiation	[113]
miR-139-3p ↑	Microgravity	*ELK1* ↓	Suppresses osteoblast differentiation	[114]
miR-140-5p ↑	hMSC	*BMP2* ↓	Regulates osteoblast differentiation	[115]
miR-146a ↑	hMSCs	*JMJD3*, *RUNX2* ↓	Inhibits osteoblast differentiation	[116]
miR-181a-5p ↑	hBMSCs cells	*ALP*, *OPN*, *RUNX2* ↓	Regulates osteoblast differentiation	[117]
miR-181a-5p↑	Blood serum	*BMP3* ↓	Inhibits osteoblast differentiation	[118]
miR194-5p ↑	Plasma from patients with osteoporotic	*WNT5a* ↓	Suppresses osteogenic differentiation	[119]
miR-206 ↑	hBMSCs	*EIF3*↓	Promoting proliferation and differentiation and inhibiting apoptosis	[120]
miR-210 ↑	Human umbilical cord blood (HUCB)-derived mesenchymal stem cells (MSCs)	*RUNX2*, *ALP* ↑	Regulates osteoblast differentiation	[121]
miR-214 ↑	Human	*MAPK* ↓ *ATF4* ↓ *OSX* ↓	Upregulation of miR-214 could efficiently promote bone marrow mesenchymal stem cell (BMSC) differentiation and reduce osteogenic differentiation	[122]
miR-218 ↑	hBMSCs	*RUNX2*, *ALP* ↑, *TOB1*, *DKK2*, *SFRP2*, *SOST* ↓	Regulates mechanisms of osteogenic differentiation	[123]
miR-223 ↑	hBMSCs	*DHRS3*	Promotes the proliferation and differentiation of osteoblasts	[124]
miR-339 ↑	Bone marrow mesenchymal stem cells	*DLX5* ↓	Upregulation of miR-339 reduces osteogenic differentiation by silencing the action of DLX5, in BMSCs	[125]
miR-449b-5p ↑	hBMSCs	*SATB2* ↓	Suppresses osteoblast differentiation	[126]
miR-486-5p ↑	hBMSCs	*COL1*, *RUNX2*, *ALP* and *BMP* ↓	Promotes the proliferation and differentiation of osteoblasts	[127]
miR-1224-5p ↑	Plasma from patients with osteoporotic fractures, bone marrow-derived macrophages, bone marrow mesenchymal stem cells, and osteoblast precursor cells	*ADCY2*, *NFATc1*, C-*FOS*, *SRC*, *ACP5*, *CTSK* ↓	Promotes the expression of signaling pathways involved in osteoblastogenesis and suppresses osteoclastogenesis activity	[128]
miR-23a ↑ miR-27a ↑ miR-24-2 ↑	Human primary fetal ROB	*RUNX2* ↓ *PPARg* ↓ *RUNX2* ↓	Regulates the osteoblast differentiation program	[129]
miR-145 ↑ miR-34c ↑	hMSC	*CBFB* ↓	Regulates mechanisms of osteogenic differentiation	[130]
miR-135 ↑ miR-203 ↑ miR-231 ↑	Biopsies derived from primary tumors and bone metastases of patients with breast cancer	*ROCK1*, *CD44*, *PTK2* ↓ *ROCK1*, *CD44*, *PTK2* ↓ *CCL7*, *CXCL12* ↓	Inhibits the migration and proliferation of osteoblasts	[131]
miR-30a-3p/5p ↑ miR-30a-3p/5p ↑ miR-194-3p/5p ↓ miR-27b-3p/5p ↓	Serum samples from patients with osteoporosis	*ROCK1*, *ALDH2*, *SOS2* ↑ *TGFB1* ↓ *NCF2* ↑ *ROCK1*, *CXCL16*, *IFNAR1* ↑	Suppress osteoclast survival and promotes the activity of osteoblasts	[132]
miR-29b-3p ↓ miR-550a-3p ↓ miR-324-3p ↓	Serum samples from patients with osteoporosis	Not mentioned	Related to the development of osteoporosis; observed to be down-expressed in patients receiving antiresorptive therapy, and proposed as biomarkers for the diagnosis of OP	[133]
miR-194-5p ↑ miR-454-3p ↑ miR-151a-3p ↑ miR-590-5p ↑ miR-1972 ↓	Mononuclear cells from postmenopausal women with osteopenia and with osteoporosis	*IRF8* ↓ *PTEN* ↓ *PTGES2* ↓ *ALDH7A1*, *PRDM1*, *PLAG1* ↓ *CDK6*, *PRDM1*, *BNIP3L* ↑	Promotes the expression of signaling pathways involved in osteoblastogenesis and suppresses osteoclastogenesis activity	[134]
miR-23a ↑ miR-30c ↑ miR-34c ↑ miR-133 ↑ miR-135a ↑ miR-137 ↑ miR-204 ↑ miR-205 ↑ miR-217 ↑ miR-218 ↑ miR-338 ↑	Different types of human mesenchymal cells	*RUNX2* ↓	Inhibits osteoblast differentiation	[135]
miR-149 ↑ miR-221 ↑ miR-628-3p ↑ miR-654-5p ↑	Fracture samples from patients with atrophic nonunion	*RUNX2*, *COL1A1*, OC ↓	Regulates mechanisms of osteogenic differentiation	[137]
Let-7b ↓ miR-220b ↓ miR-513a-3p ↓ miR-551a ↓ miR-576-5p ↓ miR-1236 ↓ vkshv-miR-K12-6-5p ↓	Fracture samples from patients with atrophic nonunion	*RUNX2* ↑	Regulates mechanisms of osteogenic differentiation	[137]

↑ upregulated; ↓ downregulated; hBMSCs: human bone marrow stem cells; hMSC: human mesenquimal stem cells.

**Table 3:** Summary of miRNAs in humans and their targets, expressions, and effects on osteocyte differentiation.

miRNA ID	Study Model	Target Genes	Effects on Bone Remodeling	Reference
miR-145-5p/β ↓	Bone biopsies of patients with adolescent idiopathic scoliosis	*CTNNB1* ↑	Restores the activity of osteocytes	[142]
miR-342 ↑	Human bone marrow stromal cells	*COL1A2* ↓	Inhibits the differentiation of bone marrow stromal cells	[143]
miR-541 ↑	Human bone marrow stromal cells	*OPN* ↓	Inhibits osteogenesis	[144]
miR-21 ↑	Not mentioned	*ERK*, *p38*, *STAT3* ↓	In women, reduces osteocyte viability in men, increases osteocyte viability	[145]
miR-23a ↑ miR-27a ↑ miR-24-2 ↑	Mouse osteoblasts	*TGF*-β, *Sost* ↓	Promotes osteocyte differentiation	[146]
miR-218 ↑	Ocy454/IDG-SW3	*Sost* ↓	Inhibits osteogenesis	[147]
miR-199a-3p ↑	C57BL/6 mice MLO-Y4 osteocytic cells	*IGF*-1, *LC3*-II ↓	regulates osteocyte autophagy through estrogen	[148]
miR-29b ↑ miR-497 ↑ miR-195 ↑ miR-30a ↑	IDG-SW3 osteocytic cells	*SEMA3* ↓	Reduces bone resorption	[149]
miR-181b-5p ↑	MLO-Y4 osteocytic cells	*PTEN*, *AKT* ↓	Induces osteogenic differentiation in humans	[150]

↑ miRNAs are upregulated; ↓ miRNAs are downregulated.

**Table 4:** Summary of miRNAs in humans and their targets, expressions, and effects on osteoporosis.

miRNA ID	Study Model	Target Genes	Effects on Bone Remodeling	Reference
miR-214 ↑	Human MSCs	*ALP*, *COL1A1*, *OCN*, *OPN* ↓	Overexpression of miR-214 promoted osteoporosis	[158]
miR-31 ↑	Human MSCs	*SATB2*, *RUNX2* ↓	Overexpression of miR-31 promoted osteoporosis	[106]
miR-449-b-5p ↑	Human MSCs	*SATB2*, *RUNX2*, *ALP*, *OCN* ↓	Overexpression of miR-449-b-5p decreased during osteogenic differentiation.	[159]
Let7d-5p ↑ Let7e-5p ↑ miR-30d-5p ↑ miR-30e-5p ↑ miR-126-3p ↑ miR-148a ↑ miR-199a-3p ↑ miR-423-5p ↑ miR-574-5p ↑	Serum	Not mentioned	Negative correlation with low BMD	[160]
miR-27a ↑	Human MSCs	*MEF2C* ↓	miR-27a decreased bone formation	[161]
miR-96 ↑	Serum	*OSX* ↓	miR-96 decreased bone formation	[162]
miR-122-5p ↑ miR-4516 ↑	Serum	*BMP2K*, *FSHB*, *IGF1R*, *PTHLH*, *RUNX2*, *SPARC*, *TSC22D3*, *VDR* ↓	Associated with fragility fracture	[163]
miR-144-3p ↑	Monocytes	*TRAP*, *CTSK*, *NFATC*, *CKK8* ↓	Involved in osteoporosis	[164]
miR-203 ↑	Serum	*DKK1* ↓	miR-203 decreased bone formation	[165]
miR-338 ↑	Serum	*RUNX2*, *SOX4* ↓	miR-338 decreased bone formation	[166]
miR-579-3p ↑	Serum	*SIRT1* ↓	Inhibits osteogenic differentiation	[167]
miR-410 ↑	PBMCs	*BMP2* ↓	Participates in postmenopausal osteoporosis	[168]

↑ miRNAs are upregulated; ↓ miRNAs are downregulated.

## Data Availability

Not applicable.
